# [*N*,*N*-Bis(diphenyl­phosphan­yl)benzyl­amine-κ^2^ *P*,*P*′]dichloridonickel(II) dichloro­methane monosolvate

**DOI:** 10.1107/S1600536811042759

**Published:** 2011-10-22

**Authors:** Bang-Shao Yin, Tian-Bao Li, Ming-Sheng Yang

**Affiliations:** a College of Chemistry and Chemical Engineering, Hunan Normal University, Changsha, Hunan 410081, People’s Republic of China

## Abstract

In the title solvated complex, [NiCl_2_(C_31_H_27_NP_2_)]·CH_2_Cl_2_, the Ni^2+^ ion is coordinated by two chloride ions and two P atoms of the chelating *N*,*N*-bis­(diphenyl­phosphan­yl)benzyl ligand to generate a strongly distorted *cis*-NiCl_2_P_2_ square-planar geometry for the metal ion. In the crystal, the components are linked by C—H⋯Cl inter­actions.

## Related literature

For details of the synthesis, see: Sun *et al.* (2006 ▶). For a related structure, see: Yin *et al.* (2011 ▶).
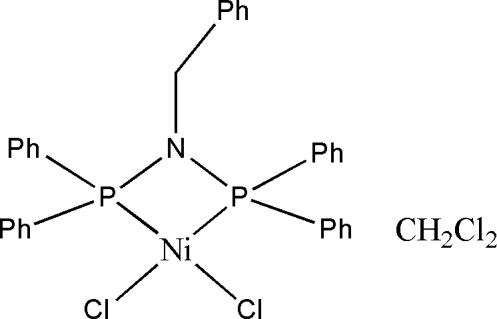


## Experimental

### 

#### Crystal data

[NiCl_2_(C_31_H_27_NP_2_)]·CH_2_Cl_2_
*M* *_r_* = 690.01
Monoclinic, 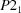

*a* = 11.074 (6) Å
*b* = 8.906 (5) Å
*c* = 15.814 (8) Å
β = 91.815 (12)°
*V* = 1558.9 (14) Å^3^
*Z* = 2
Mo *K*α radiation
μ = 1.09 mm^−1^
*T* = 113 K
0.40 × 0.18 × 0.14 mm

#### Data collection

Rigaku Saturn724 CCD diffractometer
Absorption correction: multi-scan (*CrystalClear*; Rigaku/MSC, 2005 ▶) *T*_min_ = 0.669, *T*_max_ = 0.862
13329 measured reflections
5495 independent reflections
4621 reflections with *I* > 2σ(*I*)
*R*_int_ = 0.042

#### Refinement

*R*[*F* ^2^ > 2σ(*F* ^2^)] = 0.026
*wR*(*F* ^2^) = 0.049
*S* = 0.93
5495 reflections
361 parameters
1 restraint
H-atom parameters constrained
Δρ_max_ = 0.25 e Å^−3^
Δρ_min_ = −0.28 e Å^−3^
Absolute structure: Flack (1983 ▶), 3412 Friedel pairs
Flack parameter: −0.024 (10)

### 

Data collection: *CrystalClear* (Rigaku/MSC, 2005 ▶); cell refinement: *CrystalClear*; data reduction: *CrystalClear*; program(s) used to solve structure: *SHELXS97* (Sheldrick, 2008 ▶); program(s) used to refine structure: *SHELXL97* (Sheldrick, 2008 ▶); molecular graphics: *SHELXTL* (Sheldrick, 2008 ▶); software used to prepare material for publication: *CrystalStructure* (Rigaku/MSC, 2005 ▶).

## Supplementary Material

Crystal structure: contains datablock(s) global, I. DOI: 10.1107/S1600536811042759/hb6452sup1.cif

Structure factors: contains datablock(s) I. DOI: 10.1107/S1600536811042759/hb6452Isup2.hkl

Additional supplementary materials:  crystallographic information; 3D view; checkCIF report

## Figures and Tables

**Table:** 

Ni1—P2	2.1244 (11)
Ni1—P1	2.1349 (12)
Ni1—Cl2	2.1994 (12)
Ni1—Cl1	2.2031 (12)

**Table:** 

P2—Ni1—P1	73.64 (5)
P2—Ni1—Cl2	93.79 (5)
P1—Ni1—Cl2	167.11 (3)
P2—Ni1—Cl1	167.91 (4)
P1—Ni1—Cl1	94.29 (4)
Cl2—Ni1—Cl1	98.29 (5)

**Table 2:** Hydrogen-bond geometry (Å, °)

*D*—H⋯*A*	*D*—H	H⋯*A*	*D*⋯*A*	*D*—H⋯*A*
C15—H15⋯Cl2^i^	0.95	2.72	3.626 (4)	160
C22—H22⋯Cl2^ii^	0.95	2.69	3.485 (4)	142
C25—H25*A*⋯Cl2^iii^	0.99	2.79	3.737 (4)	159
C32—H32*B*⋯Cl1	0.99	2.68	3.522 (4)	143

Symmetry codes: (i) 
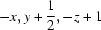
; (ii) 
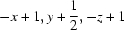
; (iii) 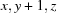.

**Fig. 1.**
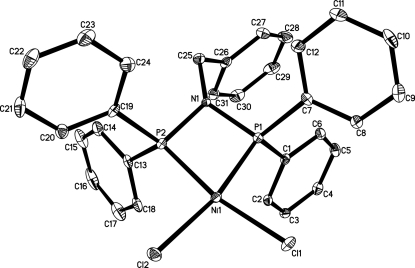
The molecular structure of (I). Displacement ellipsoids are drawn at the 30% probability level and H atoms are shown as small spheres of arbitrary radius.

**Fig. 2.**
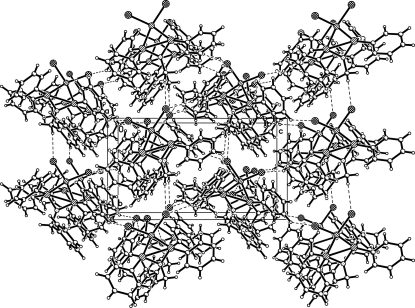
The packing for (I).

## supplementary crystallographic information

### Experimental

The title complex, (I), was prepared according to the literature procedures (Sun *et al.*, 2006). Red prisms of (I) were grown from slow evaporation of dichloromethane and hexane solution at room temperature.

### Refinement

All the H atoms were positioned geometrically (C—H = 0.93–0.97 Å) and refined as riding with *U*_iso_(H) = 1.2*U*_eq_(C) or 1.5*U*_eq_(methyl C).

### Crystal data

**Table:**

[NiCl_2_(C_31_H_27_NP_2_)]·CH_2_Cl_2_	*F*(000) = 708
*M**_r_* = 690.01	*D*_x_ = 1.470 Mg m^−^^3^
Monoclinic, *P*2_1_	Mo *K*α radiation, λ = 0.71073 Å
Hall symbol: P 2yb	Cell parameters from 6160 reflections
*a* = 11.074 (6) Å	θ = 1.3–27.9°
*b* = 8.906 (5) Å	µ = 1.09 mm^−^^1^
*c* = 15.814 (8) Å	*T* = 113 K
β = 91.815 (12)°	Prism, red
*V* = 1558.9 (14) Å^3^	0.40 × 0.18 × 0.14 mm
*Z* = 2	

### Data collection

**Table:**

Rigaku Saturn724 CCD diffractometer	5495 independent reflections
Radiation source: rotating anode	4621 reflections with *I* > 2σ(*I*)
multilayer	*R*_int_ = 0.042
Detector resolution: 14.22 pixels mm^-1^	θ_max_ = 25.0°, θ_min_ = 1.3°
ω and φ scans	*h* = −12→13
Absorption correction: multi-scan (*CrystalClear*; Rigaku/MSC, 2005)	*k* = −10→10
*T*_min_ = 0.669, *T*_max_ = 0.862	*l* = −18→18
13329 measured reflections	

### Refinement

**Table:**

Refinement on *F*^2^	Secondary atom site location: difference Fourier map
Least-squares matrix: full	Hydrogen site location: inferred from neighbouring sites
*R*[*F*^2^ > 2σ(*F*^2^)] = 0.026	H-atom parameters constrained
*wR*(*F*^2^) = 0.049	*w* = 1/[σ^2^(*F*_o_^2^) + (0.*P*)^2^] where *P* = (*F*_o_^2^ + 2*F*_c_^2^)/3
*S* = 0.93	(Δ/σ)_max_ = 0.001
5495 reflections	Δρ_max_ = 0.25 e Å^−^^3^
361 parameters	Δρ_min_ = −0.28 e Å^−^^3^
1 restraint	Absolute structure: Flack (1983), xxx Friedel pairs
Primary atom site location: structure-invariant direct methods	Flack parameter: −0.024 (10)

### Special details

**Table:**

Geometry. All e.s.d.'s (except the e.s.d. in the dihedral angle between two l.s. planes) are estimated using the full covariance matrix. The cell e.s.d.'s are taken into account individually in the estimation of e.s.d.'s in distances, angles and torsion angles; correlations between e.s.d.'s in cell parameters are only used when they are defined by crystal symmetry. An approximate (isotropic) treatment of cell e.s.d.'s is used for estimating e.s.d.'s involving l.s. planes.
Refinement. Refinement of *F*^2^ against ALL reflections. The weighted *R*-factor *wR* and goodness of fit *S* are based on *F*^2^, conventional *R*-factors *R* are based on *F*, with *F* set to zero for negative *F*^2^. The threshold expression of *F*^2^ > σ(*F*^2^) is used only for calculating *R*-factors(gt) *etc*. and is not relevant to the choice of reflections for refinement. *R*-factors based on *F*^2^ are statistically about twice as large as those based on *F*, and *R*- factors based on ALL data will be even larger.

### Fractional atomic coordinates and isotropic or equivalent isotropic displacement parameters (Å^2^)

**Table:**

	*x*	*y*	*z*	*U*_iso_*/*U*_eq_	
Ni1	0.24520 (3)	0.12907 (4)	0.25187 (2)	0.01363 (9)	
P1	0.19289 (7)	0.34210 (8)	0.20080 (5)	0.01374 (18)	
P2	0.18168 (7)	0.25751 (9)	0.35457 (5)	0.01453 (18)	
Cl1	0.30331 (7)	0.03925 (8)	0.12990 (5)	0.02271 (19)	
Cl2	0.27702 (7)	−0.07305 (8)	0.32932 (5)	0.02193 (19)	
N1	0.1629 (2)	0.4177 (2)	0.29700 (14)	0.0119 (5)	
C1	0.0598 (2)	0.3345 (3)	0.13067 (17)	0.0143 (7)	
C2	−0.0161 (3)	0.2103 (3)	0.13591 (18)	0.0173 (7)	
H2	0.0040	0.1305	0.1737	0.021*	
C3	−0.1213 (3)	0.2036 (3)	0.08575 (18)	0.0183 (7)	
H3	−0.1734	0.1194	0.0899	0.022*	
C4	−0.1506 (3)	0.3180 (3)	0.03001 (18)	0.0201 (8)	
H4	−0.2224	0.3125	−0.0043	0.024*	
C5	−0.0747 (3)	0.4409 (4)	0.02447 (18)	0.0212 (8)	
H5	−0.0949	0.5197	−0.0139	0.025*	
C6	0.0308 (3)	0.4503 (3)	0.07432 (17)	0.0181 (7)	
H6	0.0826	0.5348	0.0700	0.022*	
C7	0.3021 (2)	0.4579 (3)	0.14861 (18)	0.0154 (7)	
C8	0.3364 (3)	0.4166 (3)	0.06774 (18)	0.0205 (7)	
H8	0.2981	0.3342	0.0397	0.025*	
C9	0.4265 (3)	0.4961 (4)	0.0282 (2)	0.0287 (9)	
H9	0.4512	0.4664	−0.0263	0.034*	
C10	0.4804 (3)	0.6176 (4)	0.06764 (19)	0.0305 (8)	
H10	0.5423	0.6711	0.0404	0.037*	
C11	0.4441 (3)	0.6628 (3)	0.1479 (2)	0.0314 (9)	
H11	0.4799	0.7483	0.1745	0.038*	
C12	0.3564 (3)	0.5823 (3)	0.18788 (19)	0.0233 (8)	
H12	0.3326	0.6116	0.2427	0.028*	
C13	0.0374 (3)	0.2126 (3)	0.39800 (18)	0.0168 (7)	
C14	−0.0082 (3)	0.3010 (3)	0.46292 (19)	0.0241 (8)	
H14	0.0422	0.3739	0.4901	0.029*	
C15	−0.1253 (3)	0.2833 (4)	0.4876 (2)	0.0346 (10)	
H15	−0.1559	0.3449	0.5311	0.041*	
C16	−0.1986 (3)	0.1755 (4)	0.4491 (2)	0.0406 (11)	
H16	−0.2801	0.1654	0.4654	0.049*	
C17	−0.1546 (3)	0.0827 (4)	0.3874 (2)	0.0372 (10)	
H17	−0.2047	0.0070	0.3626	0.045*	
C18	−0.0357 (3)	0.1007 (3)	0.36136 (18)	0.0237 (8)	
H18	−0.0048	0.0369	0.3190	0.028*	
C19	0.2807 (2)	0.2932 (3)	0.44522 (18)	0.0157 (7)	
C20	0.2719 (3)	0.2011 (3)	0.51609 (19)	0.0204 (8)	
H20	0.2116	0.1254	0.5172	0.024*	
C21	0.3513 (3)	0.2202 (4)	0.5850 (2)	0.0292 (9)	
H21	0.3448	0.1582	0.6335	0.035*	
C22	0.4409 (3)	0.3307 (4)	0.5829 (2)	0.0299 (9)	
H22	0.4951	0.3440	0.6300	0.036*	
C23	0.4504 (3)	0.4198 (4)	0.51283 (19)	0.0273 (8)	
H23	0.5117	0.4944	0.5118	0.033*	
C24	0.3714 (3)	0.4025 (3)	0.4430 (2)	0.0228 (8)	
H24	0.3791	0.4642	0.3945	0.027*	
C25	0.1090 (3)	0.5631 (3)	0.32274 (18)	0.0194 (7)	
H25A	0.1721	0.6418	0.3215	0.023*	
H25B	0.0828	0.5543	0.3818	0.023*	
C26	0.0019 (3)	0.6127 (3)	0.26724 (17)	0.0175 (7)	
C27	0.0136 (3)	0.7352 (3)	0.2139 (2)	0.0238 (8)	
H27	0.0882	0.7878	0.2130	0.029*	
C28	−0.0836 (3)	0.7818 (3)	0.1614 (2)	0.0301 (9)	
H28	−0.0755	0.8668	0.1257	0.036*	
C29	−0.1912 (3)	0.7035 (3)	0.1619 (2)	0.0311 (9)	
H29	−0.2568	0.7334	0.1255	0.037*	
C30	−0.2037 (3)	0.5807 (3)	0.2156 (2)	0.0283 (9)	
H30	−0.2783	0.5279	0.2161	0.034*	
C31	−0.1081 (3)	0.5351 (3)	0.26821 (18)	0.0214 (7)	
H31	−0.1171	0.4515	0.3048	0.026*	
Cl3	0.64281 (7)	0.01513 (9)	0.20683 (5)	0.0317 (2)	
Cl4	0.54852 (8)	0.27948 (10)	0.29145 (6)	0.0474 (3)	
C32	0.5825 (3)	0.1997 (3)	0.1931 (2)	0.0323 (9)	
H32A	0.6420	0.2636	0.1646	0.039*	
H32B	0.5083	0.1951	0.1567	0.039*	

### Atomic displacement parameters (Å^2^)

**Table:**

	*U*^11^	*U*^22^	*U*^33^	*U*^12^	*U*^13^	*U*^23^
Ni1	0.0163 (2)	0.01266 (18)	0.01190 (18)	0.00101 (17)	−0.00072 (14)	−0.00153 (18)
P1	0.0160 (5)	0.0132 (4)	0.0121 (4)	−0.0003 (4)	0.0012 (3)	−0.0006 (4)
P2	0.0177 (5)	0.0142 (4)	0.0116 (4)	−0.0007 (4)	0.0006 (3)	−0.0011 (4)
Cl1	0.0259 (5)	0.0253 (5)	0.0169 (4)	0.0040 (4)	−0.0002 (3)	−0.0083 (4)
Cl2	0.0248 (5)	0.0170 (4)	0.0236 (5)	0.0030 (3)	−0.0035 (4)	0.0038 (4)
N1	0.0175 (14)	0.0081 (13)	0.0103 (13)	0.0027 (11)	0.0027 (10)	−0.0010 (11)
C1	0.0135 (17)	0.0176 (17)	0.0119 (16)	−0.0006 (14)	0.0028 (13)	−0.0020 (14)
C2	0.0253 (19)	0.0152 (17)	0.0114 (17)	0.0023 (15)	0.0010 (14)	0.0018 (14)
C3	0.0214 (19)	0.0157 (17)	0.0179 (18)	−0.0037 (14)	0.0043 (14)	−0.0026 (15)
C4	0.0150 (18)	0.029 (2)	0.0166 (18)	0.0008 (15)	−0.0008 (14)	−0.0015 (15)
C5	0.0202 (19)	0.025 (2)	0.0181 (19)	0.0034 (15)	0.0003 (14)	0.0061 (17)
C6	0.0200 (18)	0.0188 (18)	0.0156 (18)	−0.0011 (14)	0.0014 (14)	0.0023 (15)
C7	0.0121 (17)	0.0153 (17)	0.0188 (18)	0.0012 (13)	0.0019 (13)	0.0045 (14)
C8	0.0236 (19)	0.0201 (18)	0.0181 (18)	−0.0043 (15)	0.0032 (14)	−0.0001 (16)
C9	0.031 (2)	0.035 (2)	0.0209 (19)	0.0001 (18)	0.0068 (16)	0.0049 (17)
C10	0.024 (2)	0.034 (2)	0.034 (2)	−0.0086 (18)	0.0076 (16)	0.014 (2)
C11	0.037 (2)	0.021 (2)	0.036 (2)	−0.0121 (16)	0.0040 (17)	−0.0004 (17)
C12	0.0243 (19)	0.0218 (19)	0.0239 (19)	−0.0066 (15)	0.0046 (15)	−0.0028 (15)
C13	0.0148 (18)	0.0208 (18)	0.0149 (18)	−0.0016 (14)	0.0012 (13)	0.0071 (14)
C14	0.028 (2)	0.025 (2)	0.0193 (18)	0.0026 (15)	0.0048 (15)	0.0066 (16)
C15	0.032 (2)	0.037 (2)	0.036 (2)	0.0167 (19)	0.0170 (18)	0.020 (2)
C16	0.018 (2)	0.060 (3)	0.043 (2)	0.0053 (19)	0.0058 (18)	0.034 (2)
C17	0.028 (2)	0.045 (3)	0.038 (2)	−0.0170 (18)	−0.0158 (17)	0.020 (2)
C18	0.0251 (19)	0.027 (2)	0.0181 (18)	−0.0063 (16)	−0.0027 (14)	0.0112 (16)
C19	0.0135 (17)	0.0214 (18)	0.0120 (17)	0.0040 (14)	−0.0012 (13)	−0.0058 (14)
C20	0.0190 (19)	0.0239 (19)	0.0183 (18)	−0.0021 (15)	0.0012 (14)	0.0015 (15)
C21	0.030 (2)	0.042 (2)	0.0150 (19)	0.0005 (18)	−0.0032 (15)	0.0080 (17)
C22	0.021 (2)	0.047 (2)	0.021 (2)	−0.0005 (18)	−0.0074 (15)	−0.0071 (19)
C23	0.0164 (19)	0.037 (2)	0.029 (2)	−0.0076 (16)	−0.0019 (15)	−0.0077 (19)
C24	0.0196 (19)	0.0245 (19)	0.025 (2)	−0.0017 (15)	0.0067 (15)	−0.0014 (16)
C25	0.0249 (19)	0.0153 (17)	0.0181 (18)	0.0019 (14)	0.0003 (14)	−0.0012 (15)
C26	0.0205 (17)	0.0158 (16)	0.0163 (16)	0.0071 (15)	0.0014 (13)	−0.0047 (15)
C27	0.026 (2)	0.0163 (18)	0.029 (2)	−0.0017 (15)	0.0004 (15)	0.0013 (16)
C28	0.038 (2)	0.0154 (18)	0.037 (2)	0.0041 (17)	−0.0042 (17)	0.0028 (17)
C29	0.030 (2)	0.025 (2)	0.038 (2)	0.0141 (16)	−0.0088 (17)	−0.0097 (18)
C30	0.018 (2)	0.033 (2)	0.034 (2)	−0.0011 (15)	0.0008 (16)	−0.0097 (18)
C31	0.0222 (19)	0.0209 (18)	0.0214 (18)	−0.0013 (15)	0.0068 (14)	−0.0028 (16)
Cl3	0.0338 (5)	0.0281 (5)	0.0328 (5)	0.0003 (4)	−0.0035 (4)	0.0001 (4)
Cl4	0.0495 (7)	0.0551 (7)	0.0377 (6)	0.0179 (5)	0.0050 (5)	−0.0094 (5)
C32	0.036 (2)	0.030 (2)	0.031 (2)	0.0039 (17)	0.0026 (17)	−0.0002 (18)

### Geometric parameters (Å, °)

**Table:**

Ni1—P2	2.1244 (11)	C14—H14	0.9500
Ni1—P1	2.1349 (12)	C15—C16	1.386 (5)
Ni1—Cl2	2.1994 (12)	C15—H15	0.9500
Ni1—Cl1	2.2031 (12)	C16—C17	1.380 (4)
P1—N1	1.706 (2)	C16—H16	0.9500
P1—C7	1.808 (3)	C17—C18	1.401 (4)
P1—C1	1.817 (3)	C17—H17	0.9500
P1—P2	2.5525 (16)	C18—H18	0.9500
P2—N1	1.702 (2)	C19—C20	1.395 (4)
P2—C13	1.803 (3)	C19—C24	1.400 (4)
P2—C19	1.805 (3)	C20—C21	1.389 (4)
N1—C25	1.488 (3)	C20—H20	0.9500
C1—C2	1.393 (4)	C21—C22	1.399 (4)
C1—C6	1.394 (4)	C21—H21	0.9500
C2—C3	1.390 (4)	C22—C23	1.369 (4)
C2—H2	0.9500	C22—H22	0.9500
C3—C4	1.379 (4)	C23—C24	1.395 (4)
C3—H3	0.9500	C23—H23	0.9500
C4—C5	1.384 (4)	C24—H24	0.9500
C4—H4	0.9500	C25—C26	1.519 (4)
C5—C6	1.391 (4)	C25—H25A	0.9900
C5—H5	0.9500	C25—H25B	0.9900
C6—H6	0.9500	C26—C27	1.387 (4)
C7—C8	1.395 (4)	C26—C31	1.401 (4)
C7—C12	1.397 (4)	C27—C28	1.401 (4)
C8—C9	1.389 (4)	C27—H27	0.9500
C8—H8	0.9500	C28—C29	1.381 (4)
C9—C10	1.376 (4)	C28—H28	0.9500
C9—H9	0.9500	C29—C30	1.395 (4)
C10—C11	1.402 (4)	C29—H29	0.9500
C10—H10	0.9500	C30—C31	1.387 (4)
C11—C12	1.378 (4)	C30—H30	0.9500
C11—H11	0.9500	C31—H31	0.9500
C12—H12	0.9500	Cl3—C32	1.785 (3)
C13—C18	1.399 (4)	Cl4—C32	1.761 (3)
C13—C14	1.401 (4)	C32—H32A	0.9900
C14—C15	1.375 (4)	C32—H32B	0.9900
			
P2—Ni1—P1	73.64 (5)	C13—C14—H14	119.7
P2—Ni1—Cl2	93.79 (5)	C14—C15—C16	120.0 (3)
P1—Ni1—Cl2	167.11 (3)	C14—C15—H15	120.0
P2—Ni1—Cl1	167.91 (4)	C16—C15—H15	120.0
P1—Ni1—Cl1	94.29 (4)	C17—C16—C15	120.7 (3)
Cl2—Ni1—Cl1	98.29 (5)	C17—C16—H16	119.7
N1—P2—Ni1	94.60 (9)	C15—C16—H16	119.7
N1—P1—Ni1	94.10 (9)	C16—C17—C18	119.7 (3)
P2—N1—P1	97.02 (12)	C16—C17—H17	120.1
N1—P1—C7	109.50 (13)	C18—C17—H17	120.1
N1—P1—C1	112.46 (12)	C13—C18—C17	119.8 (3)
C7—P1—C1	106.49 (14)	C13—C18—H18	120.1
C7—P1—Ni1	120.15 (10)	C17—C18—H18	120.1
C1—P1—Ni1	113.74 (10)	C20—C19—C24	119.8 (3)
N1—P2—C13	107.14 (13)	C20—C19—P2	118.6 (2)
N1—P2—C19	109.77 (13)	C24—C19—P2	121.4 (2)
C13—P2—C19	105.05 (14)	C21—C20—C19	120.0 (3)
C13—P2—Ni1	119.37 (11)	C21—C20—H20	120.0
C19—P2—Ni1	119.62 (10)	C19—C20—H20	120.0
C25—N1—P2	128.80 (18)	C20—C21—C22	120.0 (3)
C25—N1—P1	132.73 (19)	C20—C21—H21	120.0
C2—C1—C6	119.9 (3)	C22—C21—H21	120.0
C2—C1—P1	118.2 (2)	C23—C22—C21	120.0 (3)
C6—C1—P1	121.9 (2)	C23—C22—H22	120.0
C3—C2—C1	119.8 (3)	C21—C22—H22	120.0
C3—C2—H2	120.1	C22—C23—C24	120.9 (3)
C1—C2—H2	120.1	C22—C23—H23	119.6
C4—C3—C2	120.6 (3)	C24—C23—H23	119.6
C4—C3—H3	119.7	C23—C24—C19	119.3 (3)
C2—C3—H3	119.7	C23—C24—H24	120.3
C3—C4—C5	119.5 (3)	C19—C24—H24	120.3
C3—C4—H4	120.2	N1—C25—C26	114.1 (2)
C5—C4—H4	120.2	N1—C25—H25A	108.7
C4—C5—C6	120.8 (3)	C26—C25—H25A	108.7
C4—C5—H5	119.6	N1—C25—H25B	108.7
C6—C5—H5	119.6	C26—C25—H25B	108.7
C5—C6—C1	119.4 (3)	H25A—C25—H25B	107.6
C5—C6—H6	120.3	C27—C26—C31	119.5 (3)
C1—C6—H6	120.3	C27—C26—C25	119.6 (3)
C8—C7—C12	119.4 (3)	C31—C26—C25	120.9 (3)
C8—C7—P1	118.2 (2)	C26—C27—C28	120.5 (3)
C12—C7—P1	122.3 (2)	C26—C27—H27	119.7
C9—C8—C7	119.9 (3)	C28—C27—H27	119.7
C9—C8—H8	120.0	C29—C28—C27	119.6 (3)
C7—C8—H8	120.0	C29—C28—H28	120.2
C10—C9—C8	120.3 (3)	C27—C28—H28	120.2
C10—C9—H9	119.9	C28—C29—C30	120.1 (3)
C8—C9—H9	119.9	C28—C29—H29	119.9
C9—C10—C11	120.3 (3)	C30—C29—H29	119.9
C9—C10—H10	119.9	C31—C30—C29	120.4 (3)
C11—C10—H10	119.9	C31—C30—H30	119.8
C12—C11—C10	119.5 (3)	C29—C30—H30	119.8
C12—C11—H11	120.3	C30—C31—C26	119.8 (3)
C10—C11—H11	120.3	C30—C31—H31	120.1
C11—C12—C7	120.6 (3)	C26—C31—H31	120.1
C11—C12—H12	119.7	Cl4—C32—Cl3	110.75 (18)
C7—C12—H12	119.7	Cl4—C32—H32A	109.5
C18—C13—C14	119.1 (3)	Cl3—C32—H32A	109.5
C18—C13—P2	120.5 (2)	Cl4—C32—H32B	109.5
C14—C13—P2	119.9 (2)	Cl3—C32—H32B	109.5
C15—C14—C13	120.6 (3)	H32A—C32—H32B	108.1
C15—C14—H14	119.7		
			
P2—Ni1—P1—N1	5.48 (8)	C4—C5—C6—C1	−0.2 (4)
Cl2—Ni1—P1—N1	18.5 (2)	C2—C1—C6—C5	0.6 (4)
Cl1—Ni1—P1—N1	−174.07 (8)	P1—C1—C6—C5	−177.5 (2)
P2—Ni1—P1—C7	120.86 (12)	N1—P1—C7—C8	−178.5 (2)
Cl2—Ni1—P1—C7	133.90 (18)	C1—P1—C7—C8	−56.6 (3)
Cl1—Ni1—P1—C7	−58.69 (12)	Ni1—P1—C7—C8	74.5 (3)
P2—Ni1—P1—C1	−111.26 (10)	P2—P1—C7—C8	139.8 (2)
Cl2—Ni1—P1—C1	−98.22 (19)	N1—P1—C7—C12	4.4 (3)
Cl1—Ni1—P1—C1	69.18 (10)	C1—P1—C7—C12	126.2 (3)
Cl2—Ni1—P1—P2	13.04 (17)	Ni1—P1—C7—C12	−102.7 (2)
Cl1—Ni1—P1—P2	−179.55 (4)	P2—P1—C7—C12	−37.3 (3)
P1—Ni1—P2—N1	−5.50 (8)	C12—C7—C8—C9	2.1 (4)
Cl2—Ni1—P2—N1	177.39 (9)	P1—C7—C8—C9	−175.2 (2)
Cl1—Ni1—P2—N1	−3.4 (2)	C7—C8—C9—C10	−1.5 (5)
P1—Ni1—P2—C13	107.13 (12)	C8—C9—C10—C11	−0.4 (5)
Cl2—Ni1—P2—C13	−69.98 (12)	C9—C10—C11—C12	1.6 (5)
Cl1—Ni1—P2—C13	109.3 (2)	C10—C11—C12—C7	−1.0 (5)
P1—Ni1—P2—C19	−121.36 (12)	C8—C7—C12—C11	−0.8 (5)
Cl2—Ni1—P2—C19	61.54 (12)	P1—C7—C12—C11	176.3 (2)
Cl1—Ni1—P2—C19	−119.2 (2)	N1—P2—C13—C18	101.7 (2)
Cl2—Ni1—P2—P1	−177.11 (4)	C19—P2—C13—C18	−141.6 (2)
Cl1—Ni1—P2—P1	2.13 (18)	Ni1—P2—C13—C18	−3.9 (3)
C7—P1—P2—N1	71.49 (17)	P1—P2—C13—C18	58.3 (3)
C1—P1—P2—N1	−90.16 (16)	N1—P2—C13—C14	−71.2 (3)
Ni1—P1—P2—N1	171.72 (13)	C19—P2—C13—C14	45.5 (3)
N1—P1—P2—C13	82.17 (17)	Ni1—P2—C13—C14	−176.8 (2)
C7—P1—P2—C13	153.65 (17)	P1—P2—C13—C14	−114.6 (2)
C1—P1—P2—C13	−7.99 (17)	C18—C13—C14—C15	−3.2 (4)
Ni1—P1—P2—C13	−106.11 (12)	P2—C13—C14—C15	169.8 (2)
N1—P1—P2—C19	−72.03 (17)	C13—C14—C15—C16	1.0 (5)
C7—P1—P2—C19	−0.54 (19)	C14—C15—C16—C17	1.6 (5)
C1—P1—P2—C19	−162.19 (16)	C15—C16—C17—C18	−2.0 (5)
Ni1—P1—P2—C19	99.69 (13)	C14—C13—C18—C17	2.7 (4)
N1—P1—P2—Ni1	−171.72 (13)	P2—C13—C18—C17	−170.2 (2)
C7—P1—P2—Ni1	−100.24 (13)	C16—C17—C18—C13	−0.2 (5)
C1—P1—P2—Ni1	98.12 (12)	N1—P2—C19—C20	155.3 (2)
C13—P2—N1—C25	51.5 (3)	C13—P2—C19—C20	40.4 (3)
C19—P2—N1—C25	−62.1 (3)	Ni1—P2—C19—C20	−97.1 (2)
Ni1—P2—N1—C25	174.2 (2)	P1—P2—C19—C20	−162.63 (17)
P1—P2—N1—C25	167.5 (3)	N1—P2—C19—C24	−29.8 (3)
C13—P2—N1—P1	−116.01 (13)	C13—P2—C19—C24	−144.7 (2)
C19—P2—N1—P1	130.43 (13)	Ni1—P2—C19—C24	77.8 (3)
Ni1—P2—N1—P1	6.66 (10)	P1—P2—C19—C24	12.3 (3)
C7—P1—N1—C25	62.6 (3)	C24—C19—C20—C21	1.4 (4)
C1—P1—N1—C25	−55.5 (3)	P2—C19—C20—C21	176.4 (2)
Ni1—P1—N1—C25	−173.3 (2)	C19—C20—C21—C22	−0.7 (5)
P2—P1—N1—C25	−166.7 (3)	C20—C21—C22—C23	−0.2 (5)
C7—P1—N1—P2	−130.64 (13)	C21—C22—C23—C24	0.2 (5)
C1—P1—N1—P2	111.19 (13)	C22—C23—C24—C19	0.5 (5)
Ni1—P1—N1—P2	−6.62 (10)	C20—C19—C24—C23	−1.3 (4)
N1—P1—C1—C2	−86.1 (2)	P2—C19—C24—C23	−176.1 (2)
C7—P1—C1—C2	154.0 (2)	P2—N1—C25—C26	−122.1 (3)
Ni1—P1—C1—C2	19.3 (2)	P1—N1—C25—C26	40.9 (4)
P2—P1—C1—C2	−40.4 (3)	N1—C25—C26—C27	−109.9 (3)
N1—P1—C1—C6	92.1 (2)	N1—C25—C26—C31	69.1 (3)
C7—P1—C1—C6	−27.8 (3)	C31—C26—C27—C28	0.2 (4)
Ni1—P1—C1—C6	−162.5 (2)	C25—C26—C27—C28	179.2 (3)
P2—P1—C1—C6	137.8 (2)	C26—C27—C28—C29	−1.1 (5)
C6—C1—C2—C3	−0.9 (4)	C27—C28—C29—C30	1.3 (5)
P1—C1—C2—C3	177.3 (2)	C28—C29—C30—C31	−0.7 (5)
C1—C2—C3—C4	0.8 (4)	C29—C30—C31—C26	−0.2 (4)
C2—C3—C4—C5	−0.3 (4)	C27—C26—C31—C30	0.4 (4)
C3—C4—C5—C6	0.1 (4)	C25—C26—C31—C30	−178.6 (3)

### Hydrogen-bond geometry (Å, °)

**Table:**

*D*—H···*A*	*D*—H	H···*A*	*D*···*A*	*D*—H···*A*
C15—H15···Cl2^i^	0.95	2.72	3.626 (4)	160
C22—H22···Cl2^ii^	0.95	2.69	3.485 (4)	142
C25—H25A···Cl2^iii^	0.99	2.79	3.737 (4)	159
C32—H32B···Cl1	0.99	2.68	3.522 (4)	143

Symmetry codes: (i) −*x*, *y*+1/2, −*z*+1; (ii) −*x*+1, *y*+1/2, −*z*+1; (iii) *x*, *y*+1, *z*.
